# A Systematic Review and Meta-Analysis of the Efficacy of Uterine Artery Embolization in the Treatment of Endometriosis

**DOI:** 10.1155/2022/8966063

**Published:** 2022-09-24

**Authors:** Li Ma, Bingxin Wen, Zhenhua Wen

**Affiliations:** ^1^Operating Room,the First Hospital of China Medical University, Shenyang 110001, Liaoning, China; ^2^Department of General Surgery,Jin Qiu Hospital of Liaoning, Shenyang 110016, Liaoning, China

## Abstract

**Objective:**

To compare the efficacy of uterine artery embolization (UAE) with traditional methods for treating endometriosis.

**Methods:**

The randomized controlled trials of uterine artery embolization and other medical treatments for endometriosis in PubMed, Embase, Web of Science, Cochrane Library, China Journal Full-Text Database, Wanfang Database, VIP Database, and China Biomedical Literature Database were retrieved by computer. The search time was up to June 2022. The quality of articles was evaluated by Cochrane ROB 2.0, and meta-analysis was performed by Stata15.1 software.

**Results:**

7 studies were finally included. Meta-analysis showed that the serum CA125 level after uterine artery embolization was significantly lower than that in the control group (SMD = −0.85, 95%CI (−1.12, −0.59)], and the postoperative visual analogue scale (VAS) of dysmenorrhea was significantly lower than that in the control group (SMD = −1.86, 95%CI (−2.21, −1.50)) There was no significant difference in the effective rate, FSH level, E2 level, and LH level between the two groups.

**Conclusion:**

UAE can effectively reduce the VAS score of dysmenorrhea and serum CA125 level for treating endometriosis. However, due to the limitation of the quality of included articles, more large sample size and high quality RCTs are needed to provide stronger evidence-based medicine evidence for clinical practice.

## 1. Foreword

Endometriosis (EMs) refers to inflammatory diseases in which endometrial tissue with growth function appears in the lining endometrium of the uterine cavity as well as in parts other than the uterus, affecting about 5% to 10% of women worldwide and women of reproductive age [1], and is regarded as a modern disease [2]. The main clinical manifestations are pain, mass, infertility, and symptoms of pelvic pain occurring in 70%–80% of patients [3]. Long-term management is needed to ease pain [4, 5]. At present, western medicine mainly adopts hormone therapy alone or combined surgery. Although it can slow down the development of endometriosis lesions to a certain extent, achieve the purpose of relieving the disease and inhibiting recurrence, the damage of ovarian function by surgery, the damage to the pelvic environment, as well as the unavoidable high recurrence rate after surgery and the untoward reactions existing in the process of hormone drug therapy such as irregular vaginal bleeding, menopausal symptoms, and thrombotic risk all affect its clinical efficacy and long-term clinical use. In view of its unclear pathogenesis, the efficacy of surgical treatment alone is not satisfactory, there has been a long lack of more effective treatment. In recent years, minimally invasive therapy represented by uterine artery embolization has gradually emerged. UAE has been first applied in the diagnosis and treatment of female surgical pelvic bleeding and gynecological diseases, which shows the advantages of less trauma, rapid postoperative recovery, and preservation of the uterus and exact effect and conforms to the people-oriented medical ethical concept. In this study, we systematically evaluated randomized controlled trials of UAE compared with other treatments for endometriosis in order to provide evidence-based evidence for clinicians to select reasonable surgical methods in the future.

## 2. Materials and Methods

### 2.1. Literature Retrieval

Databases in English were searched: PubMed, Embase, Web of Science, and Cochrane Library, while databases in Chinese were searched: China Journal Full-text Database, Wanfang Database, VIP Database, and China Biomedical Literature Database. The RCTs related to EMs after uterine artery embolization were retrieved from the established until June 2022. Use subject to merge search methods for free words. The search terms were *[uterine artery] [embolization endometriosis] [chocolate cyst] [Randomized]*.

### 2.2. Inclusion Criteria

- RCTs of UAE combined with other therapies for the treatment of Ems published in Chinese or English
- Patients diagnosed with endometriosis according to diagnostic criteria
- uterine artery embolization was adopted in the observation group, while other treatments were adopted in the control group

### 2.3. Exclusion Criteria

- Study on the treatment inconsistent with the research purpose between the observation group and the control group
- Articles that are published repeatedly or with incorrect data
- Articles for which outcome indicators could not be extracted

### 2.4. Risk Assessment of Literature Bias

The Cochrane ROB 2.0 [6] was used to assess the risk of publication bias from the aspects of randomization process, deviation from established interventions, lack of outcome data, measurement of results, selective reporting bias, and total bias. It was conducted independently and cross-checked by two researchers.

### 2.5. Data Extraction

Literature screening was conducted by two colleagues independently, and the data were extracted and cross-checked. When differences of opinion arose, the two researchers discussed and resolved the differences with the third researcher. The extracted contents mainly include the following:

- Basic information of the literature: author, year of publication, title, and source of the article
- The evaluation factors of bias risk were as follows: random method and blind method
- Intervention measure
- Outcome indicators

### 2.6. Statistical Analysis

Stata 15.1 software was used to process and analyze the included studies, enumeration data were expressed by relative risk (*RR*), measurement data were expressed by standardized mean difference (*SMD*), and 95%CI was used to express the results. Heterogeneity was quantitatively evaluated using *χ*^2^ test and *I*^*2*^. If there was no significant heterogeneity (*P* > 0.1, *I*^*2*^ < 50%), fixed-effect model was used for meta-analysis, while sensitivity analysis or subgroup analysis was used to investigate the source of heterogeneity. Random effect model was used for analysis when significant clinical heterogeneity was excluded. *P* < 0.05 was considered statistically significant. Egger's test was used to quantitatively detect publication bias.

## 3. Result

### 3.1. Literature Retrieval Results

**Figure 1:**
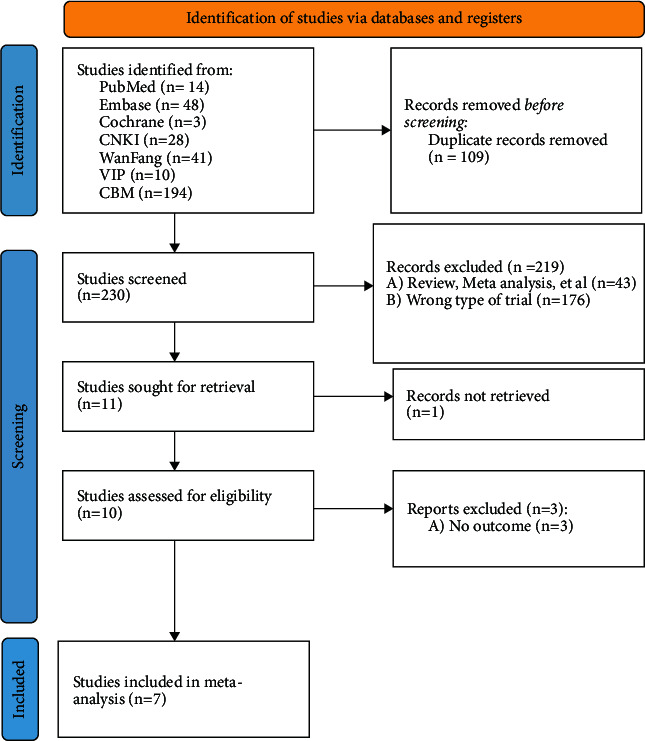
Screening plot.

A total of 338 articles were retrieved. After deduplication and preliminary screening, the remaining 230 articles were first screened, and finally, 7 articles were included. As shown in Figure 1, the basic situation of the inclusion is shown in Table 1.

### 3.2. Risk of Inclusion Study Bias

**Figure 2:**
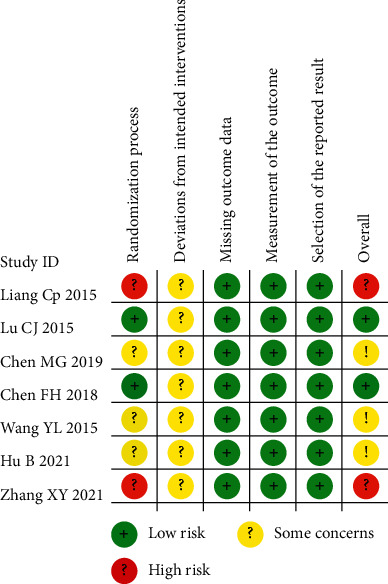
Detailed plot of bias of 7 included literature studies: based on Cochrane ROB 2.0.

**Figure 3:**
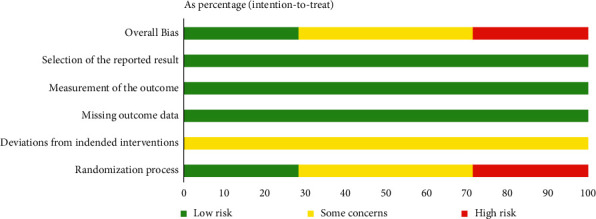
Summary plot of bias for the 7 included literature studies: based on Cochrane ROB 2.0.

7 articles with a total of 524 patients were included. Outcome indicators: total efficiency was reported in 5 articles, serum CA125 levels were measured in 3 articles, dysmenorrhea visual analogue scale was reported in 2 articles, and sex hormone levels were measured in 2 articles. 2 articles had a low risk of the randomization process, 2 articles had a high risk of the randomization process, and 3 articles only mentioned randomization, none mentioned deviation from established interventions, none lacked outcome data, had a low risk of outcome measurement, and had a low risk of selective reporting bias as shown in Figures 2 and 3.

### 3.3. Total Effective Rate

According to literature [14], it is considered to be effective when the clinical symptoms of the patient relieve or disappear, the pelvic mass shrinks or does not increase, and the clinical symptoms of the patient do not worsen after 3 months of drug discontinuation. Patient's clinical symptoms and signs were improved, or even worsened, and were considered invalid.

**Figure 4:**
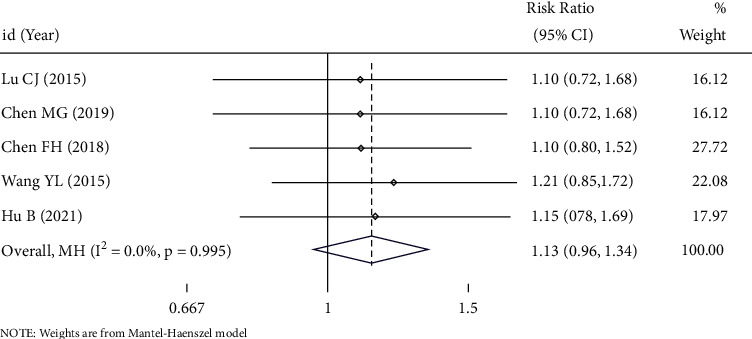
Forest plot of combined results for total effective rate.

364 patients were included in the 5 studies to compare the difference in the total effective rate between the two groups, with statistically significant heterogeneity between the literature stuides: *I*^*2*^ = 0.0%, *P*=0.995. Therefore, the fixed-effect model was used for analysis. The results showed that there was no significant difference in the effective rate of treatment of endometriosis with UAE and other treatment methods: (RR = 1.13, 95%CI (0.96, 1.34), *P* < 0.05), as shown in Figure 4.

### 3.4. Serum CA125 Level

**Figure 5:**
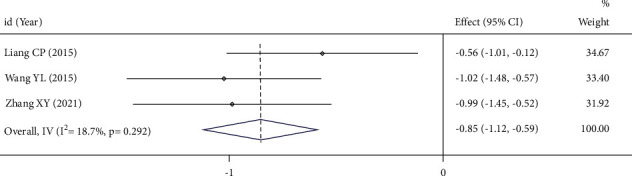
Forest plot of combined results for serum CA125 level.

244 patients were included in the 3 RCTs, with statistically significant heterogeneity between the literature stuides: *I*^*2*^ = 18.7%, *P*=0.292. Therefore, the fixed-effect model was used. Meta-analysis showed that the serum CA125 level in the UAE group was lower than that in the control group (SMD = −0.85, 95%CI (−1.12, −0.59)), *P* < 0.05, indicating that the difference was statistically significant, as shown in Figure 5.

### 3.5. VAS Score of Dysmenorrhea

**Figure 6:**
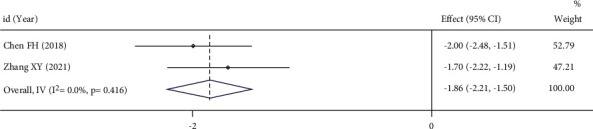
Forest plot of combined results for the VAS score of dysmenorrhea.

178 patients were included in the 2 RCTs. The heterogeneity test results showed that the components had relatively small heterogeneity: *I*^*2*^ = 0.0%, *P*=0.416. Therefore, the fixed-effect model was adopted. Meta-analysis showed that the VAS score of dysmenorrhea in the UAE group was lower than that in the control group (SMD = −1.86, 95%CI (−2.21, −1.50)), *P* < 0.05, indicating a statistically significant difference, as shown in Figure 6.

### 3.6. Sex Hormone Levels

160 patients were included in 2 literature studies. E2 level was highly heterogeneous among studies: *I*^*2*^ = 96.8%. Random effect model was used for analysis. Meta-analysis showed that there was no significant difference in E2 level between UAE and other treatment methods for endometriosis (SMD = −1.32, 95%CI (−3.33, 0.69), *P*=0.197). The results of FSH level (SMD = −0.23, 95%CI (−0.54, 0.09)) and LH level (SMD = 0.39, 95%CI (−0.50, 1.29)) showed no significant difference.

### 3.7. Publication Bias

**Figure 7:**
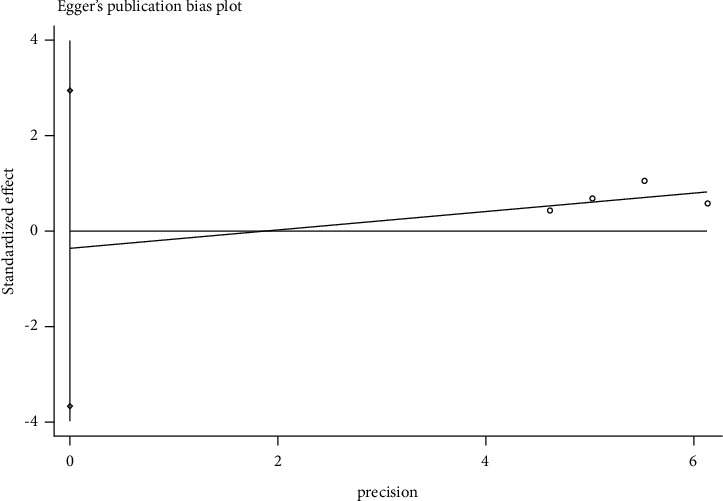
Egger's test.

The total effective rate was tested for potential publication bias by Egger's Test. The results of Egger's Test analysis are shown in Figure 7, *P*=0.752, indicating that there was no significant publication bias.

## 4. Discussion

Endometriosis is a common disease and multiple diseases in women of childbearing age, and its pathogenesis is dominated by Sampson menstrual blood reflux implantation. Endometrium reflux to the pelvic cavity requires adhesion, invasion, and vascular formation to obtain implantation growth and finally develop lesions; traits of eutopic endometrium play a decisive role, that is “eutopic endometrium determinism” [15]; other pathogeneses include metaplasia of coelomic epithelium, immune-inflammation, abnormal expression of estrogen and progesterone receptors, and genetic factors [16, 17]. Endometriosis has familial aggregation, and women with endometriosis in first-degree relatives have a relatively 7–10-fold increased risk of endometriosis. Clinically, it is usually treated with surgery or combined hormonal drugs, such as the use of nonsteroidal anti-inflammatory drugs to inhibit the synthesis of prostaglandins, inhibit lymphocyte activity and differentiation of activated T cells, reduce the stimulation of afferent nerve endings, act directly on nociceptors, and inhibit the formation and release of pain-inducing substances; oral contraceptives inhibit ovulation; highly effective progestins cause endometrial decidual-like changes, ultimately leading to endometrial atrophy, while negative feedback inhibits the hypothalamic-pituitary-ovarian axis; GnRH-a can downregulate pituitary function, cause temporary medical castration and estrogen status in the body, and can also bind to GnRH-a receptors in the periphery to inhibit the activity of eutopic and ectopic endometrial cells; gestrinone reduces ER and PR levels, reduces estrogen levels in the blood, and reduces sex hormone binding globulin levels. Lesion resection, as one of the main methods to treat endometriosis, has the characteristics of good radical effect and low recurrence rate compared with drug therapy. Laparoscopic resection, as a minimally invasive surgery, compared with traditional laparotomy, laparoscopic surgery can significantly reduce the interference with the internal environment of patients, and at the same time, it can significantly reduce the fear and tension of patients to the operation, which is conducive to the postoperative recovery of patients. However, some studies have successively found that although laparoscopic resection has more advantages compared with traditional surgery, some patients still experience recurrence and even infertility [18].

At present, with the continuous progress of interventional therapy techniques, uterine artery embolization can also be used as one of the treatment options for endometriosis. In this study, a meta-analysis of 7 included RCT studies showed that there were significant differences in dysmenorrhea VAS score and serum CA125 level, both suggested that the results of the UAE group were better than those of the control group, and serum CA125, as a common serum tumor marker, belonged to the tumor-associated antigen recognized by the monoclonal antibody OC125 for epithelial ovarian cancer and was mostly used in the diagnosis of ovarian cancer and the detection after treatment. After treatment, the CA125 level in the observation group was significantly decreased, indicating that the diseased endometrium was better improved, and the VAS score of dysmenorrhea could directly reflect the improvement of the efficacy of patients after surgery, suggesting that the patients in the observation group could relieve more pain after surgery. However, the total effective rate and serum sex hormone levels were not significantly lower than those in the control group. Zhang Xinyu et al. [13] reported the occurrence of infection, bleeding, and other adverse reactions after UAE, but there was no statistical significance. As there were few reports related to overall complications, no meta-analysis was performed. It could not confirm whether the report of this complication was related to individual differences in the condition and physician's operation level.

The limitations of this paper include the following: ① 7 randomized controlled trials included have certain risks during the randomization process, which may cause certain selectivity bias; ② no relevant English literatures were retrieved, and only the Chinese literatures that met the criteria were studied; ③ the quality of the relevant literatures published at present is low, which may have a certain impact on the reliability of the results. The impact of commonly used antibiotics, e.g., amoxicillin [19], ornidazole [20], etc., on patient treatment also needs to be investigated.

To sum up, UAE is superior to traditional treatment in the postoperative serum CA125 level and VAS score of dysmenorrhea, but it still cannot replace medication and traditional surgery. In clinic, appropriate treatment should be selected according to the specific condition of patients with endometriosis. In the future, more large-scale randomized controlled trials with higher quality and higher sample size should be conducted to further study the treatment of endometriosis with UAE.

## Figures and Tables

**Table 1:** Baseline information of the literature.

Study	Year	Age		Total		Interventions		Outcome measurements
		T	C	T	C	T	C	
Liang CP et al. [7]	2015	36.0 ± 5.0	34.0 ± 6.0	40	40	UAE	Traditional surgery	②⑤
Lu CJ et al. [8]	2015	32.2 ± 6.1		30	30	UAE	Lesion resection	①
Chen MG et al. [9]	2019	35.55 ± 3.91	36.21 ± 4.16	30	30	UAE	Traditional surgery	①②⑤
Chen FH et al. [10]	2018	37.57 ± 8.12	37.64 ± 8.20	50	48	UAE	Ibuprofen	①③
Wang YL et al. [11]	2015	32.16 ± 6.71		42	42	UAE	Ultrasound ablation	①②⑤
Hu B et al. [12]	2021	32.4 ± 11.2	31.6 ± 10.8	31	31	UAE	Traditional surgery	①②
Zhang XY et al. [13]	2021	35.85 ± 5.06	36.06 ± 5.18	43	37	UAE	Lesion resection	①②③⑤

UAE: uterine artery embolization; T: trial; C: control; ① total efficiency; ② serum CA125 level; ③ dysmenorrhea VAS score; ④ sex hormone levels (FSH, LH, and E2); ⑤ others.

## Data Availability

The data used in this study are available from the corresponding author upon request.
